# Neural network application for assessing thyroid-associated orbitopathy activity using orbital computed tomography

**DOI:** 10.1038/s41598-023-40331-1

**Published:** 2023-08-10

**Authors:** Jaesung Lee, Sanghyuck Lee, Won Jun Lee, Nam Ju Moon, Jeong Kyu Lee

**Affiliations:** 1 Department of Artificial Intelligence, Chung-Ang University, Seoul, Korea; 2 AI/ML Research Innovation Center, Chung-Ang University, Seoul, Korea; 3 Department of Ophthalmology, Chung-Ang University College of Medicine, Chung-Ang University Hospital, 102 Heukseok-Ro, Dongjak-Gu, Seoul, 06973 Korea

**Keywords:** Eye diseases, Diagnostic markers, Tomography, Software

## Abstract

This study aimed to propose a neural network (NN)-based method to evaluate thyroid-associated orbitopathy (TAO) patient activity using orbital computed tomography (CT). Orbital CT scans were obtained from 144 active and 288 inactive TAO patients. These CT scans were preprocessed by selecting eleven slices from axial, coronal, and sagittal planes and segmenting the region of interest. We devised an NN employing information extracted from 13 pipelines to assess these slices and clinical patient age and sex data for TAO activity evaluation. The proposed NN’s performance in evaluating active and inactive TAO patients achieved a 0.871 area under the receiver operating curve (AUROC), 0.786 sensitivity, and 0.779 specificity values. In contrast, the comparison models CSPDenseNet and ConvNeXt were significantly inferior to the proposed model, with 0.819 (*p* = 0.029) and 0.774 (*p* = 0.04) AUROC values, respectively. Ablation studies based on the Sequential Forward Selection algorithm identified vital information for optimal performance and evidenced that NNs performed best with three to five active pipelines. This study establishes a promising TAO activity diagnosing tool with further validation.

## Introduction

Thyroid-associated orbitopathy (TAO) is an autoimmune disorder associated with Graves’ disease (GD)^1,2^. TAO patients endure various symptoms, from dry eye and tearing to severe functional abnormalities such as visual disturbances and diplopia. These symptoms commence with eyelid and orbit inflammation that exacerbates as inflammation progresses^3^. Therefore, inflammation onset and severity evaluations are vital for designing treatment policies and predicting TAO prognosis. The clinical activity score (CAS) is the most widely used inflammatory degree assessment for TAO patients but partly relies on subjective responses^4,5^. In addition, CAS cannot sufficiently distinguish active inflammation from congestive changes in orbital soft tissue, a common severe TAO symptom. Therefore, a more objective and quantified method for evaluating orbital inflammation is imperative.

Radiological examinations have been extensively studied as potential alternatives for evaluating orbital inflammation, with computed tomography (CT) the most widely used in TAO diagnosis. Several studies certify that CT facilitates inflammation assessment in TAO patients^6,7^. However, since CT image interpretation is highly dependent on clinician experience, interpreted data may be incomplete or engender inconsistencies. Neural network (NN)-based method efficacies in eyelid and orbital disease have recently garnered attention^8–10^, and several studies have integrated NNs into radiographic image analysis to overcome these shortcomings^11–13^. Orbital image analysis using an NN can be applied for TAO diagnosis, and TAO activity evaluation using MRI has been recently reported^14^. However, an NN-based method for TAO activity evaluation using CT images, which are more widely used and cost-effective than MRI, has yet to be considered.

This study devises a new NN that evaluates TAO activity by analyzing CT images. Although TAO is more common in women, older men have worse clinical features^15^. However, it is unclear whether additional clinical information, such as age and sex, can improve NN performance. Therefore, we also strove to confirm whether NN performance could be improved by supplementing orbital CT images with clinical data.

## Results

This study included 144 active (52 men, 92 women) and 288 (41 men, 247 women) inactive TAO patients; the male subject proportion was higher in the active group than in the inactive group. The mean active TAO patient age was 46.1 ± 13.6 years, older than inactive TAO patients (p < 0.001). Table 1 organizes these characteristics.

**Table 1:** Subject characteristics.

Characteristics	Active TAO	Inactive TAO	*p-*value
Number of subjects (N)	144	288	
Age (years, mean ± SD)	46.1 ± 13.6	35.9 ± 11.7	< 0.001
Sex (male: female)	52:92	41:247	< 0.001
Clinical activity score (range)	3.4 (3–6)	0.7 (0–2)	

*TAO* thyroid-associated orbitopathy.

Table 2 summarizes the proposed and two comparative NN performance evaluations. The proposed NN’s performance in evaluating active and inactive TAO patients achieved a 0.871 area under the receiver operating curve (AUROC), 0.786 sensitivity, and 0.779 specificity values. In contrast, the comparison models CSPDenseNet and ConvNeXt were significantly inferior the proposed model, with 0.819 (*p* = 0.029) and 0.774 (*p* = 0.004) AUROC, 0.774 *(p* = 0.028*)* and 0.694 (*p* = 0.005) sensitivity, and 0.731 (*p* = 0.110) and 0.692 (*p* = 0.008) specificity values, respectively. Notably, the proposed NN significantly outperformed ConvNeXt in four additional metrics. In contrast, there was no statistically significant accuracy (p = 0.071), F1 score (*p* = 0.052), or precision (*p* = 0.069) difference between our proposed model and CSPDenseNet.

**Table 2:** Performance evaluation of the neural network models’ TAO activity assessment.

	Proposed		ConvNeXt		CSPDenseNet	
	Mean ± SD	*p*-value	Mean ± SD	*p*-value	Mean ± SD	*p*-value
AUROC	**0.871 ± 0.041**		0.774 ± 0.073	0.004	0.819 ± 0.082	0.029
Accuracy	**0.782 ± 0.044**		0.692 ± 0.072	0.006	0.735 ± 0.090	0.071
F1 score	**0.705 ± 0.055**		0.601 ± 0.079	0.005	0.656 ± 0.091	0.052
Sensitivity	**0.786 ± 0.044**		0.694 ± 0.071	0.005	0.744 ± 0.065	0.028
Specificity	**0.779 ± 0.045**		0.692 ± 0.073	0.008	0.731 ± 0.104	0.110
Precision	**0.640 ± 0.062**		0.531 ± 0.081	0.005	0.588 ± 0.101	0.069

*TAO* thyroid-associated orbitopathy. The best model was indicated in bold, as determined by paired t-tests.

We identified CT slices vital for NN performance through an ablation study that activated or deactivated input pipelines. Table 3 conveys the top five pipeline combination performances identified from our ablation study and sorts them by AUROC value. The best pipeline combination was SA_R, CO2, CO1, and AX1 with the highest value in six metrics (AUROC, 0.871; accuracy, 0.803; F1 score, 0.732; sensitivity, 0.805; specificity, 0.802; precision, 0.671). The second-best combination was CO2 and SA_R with a 0.871 AUROC.

**Table 3:** Top five AUROC-sorted pipeline combinations among the ten identified from the pipeline enable/disable procedure.

Enabled pipelines	AUROC	Accuracy	F1 score	Sensitivity	Specificity	Precision
SA_R, CO2, CO1, AX1	**0.871 ± 0.048**	**0.803 ± 0.052**	**0.732 ± 0.065**	**0.805 ± 0.054**	**0.80 2 ± 0.051**	**0.671 ± 0.071**
CO2, SA_R	0.871 ± 0.036	0.798 ± 0.029	0.724 ± 0.038	0.800 ± 0.031	0.798 ± 0.028	0.662 ± 0.043
AX3, CO3, AX6, CO1, AX4, CO2, AX2, age, SA_R, sex, SA_L	0.863 ± 0.038	0.774 ± 0.040	0.694 ± 0.049	0.772 ± 0.043	0.775 ± 0.038	0.630 ± 0.052
CO2, sex, CO1, age	0.861 ± 0.066	0.779 ± 0.075	0.702 ± 0.094	0.777 ± 0.073	0.780 ± 0.076	0.642 ± 0.107
AX3, SA_L, AX6, CO2, CO1, AX1, AX5, sex, AX2, SA_R	0.858 ± 0.036	0.777 ± 0.043	0.698 ± 0.051	0.77 7 ± 0.040	0.770 ± 0.063	0.627 ± 0.085

Significant values are in bold.

The values are expressed as the Mean ± SD. *AX1* slice with largest lens in the axial plane, *AX2* slices 3 mm above AX1 in the axial plane, *AX3* slice 3 mm below AX1 in the axial plane, *AX4* slices 7 mm above AX1 in the axial plane, *AX5* slice 7 mm below AX1 in the axial plane, *AX6* slice with the largest lacrimal gland in the axial plane, *CO1* slice with largest eyeball in the coronal plane, *CO2* 1/2 distance between the CO1 and orbit exit, *CO2* 1/3 distance between the CO1 and orbit exit, *SA_L* largest eyeball in the sagittal plane of left orbit, *SA_R* largest eyeball in the sagittal plane of right orbit.

**Figure 1:**
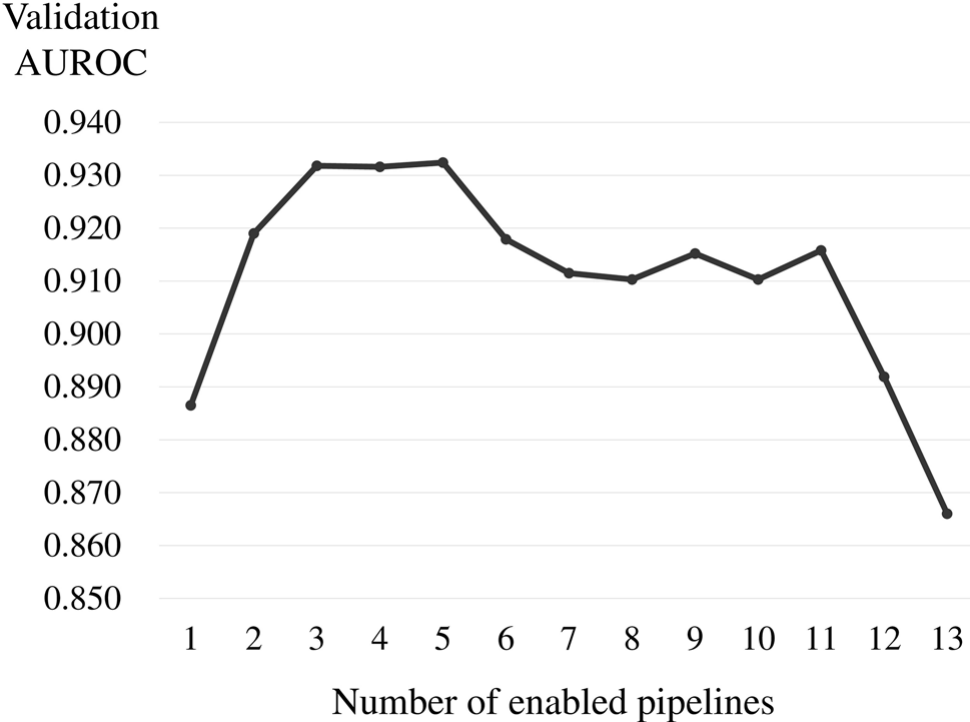
The pipeline enabling/disabling process. The y-axis represents the ten iterations for mean AUROC validation, and the x-axis represents enabled pipeline numbers. Mean AUROC validation was highest when five pipelines were enabled and lowest when all pipelines were enabled.

**Figure 2:**
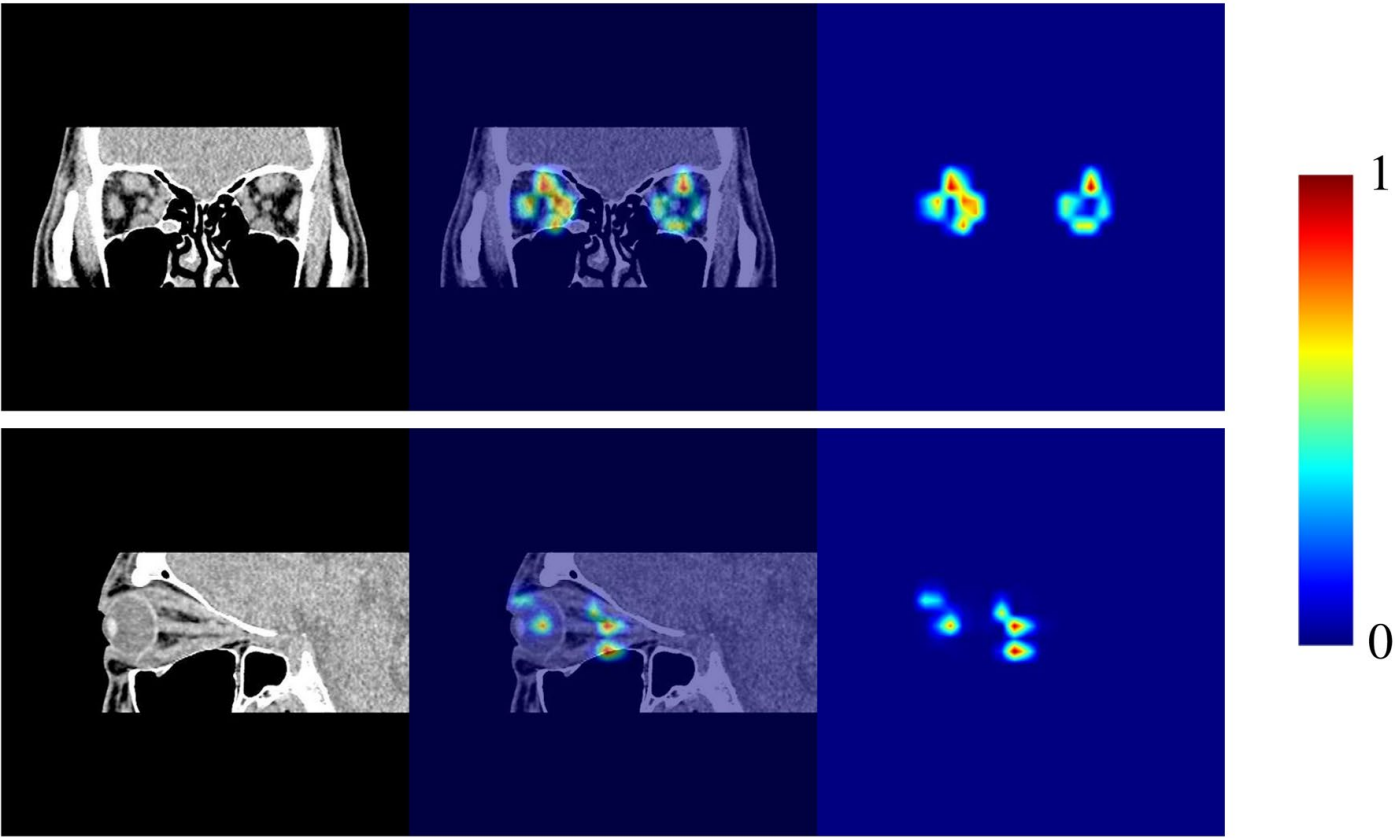
Critical diagnosis region visualization. The model with the best pipeline combination is visualized in Table 3 using Gradient-weighted Class Activation Mapping (Grad-CAM). Red signifies importance, and blue indicates insignificance. The first row is CO2 (the coronal slice located 1/2 distance between the largest eyeball and orbital exit slices), and the second is SA_R (the sagittal slice for the right eye). The first column is the original image, the second is the Grad-CAM overlay on the original image, and the third is the Grad-CAM heatmap.

The AUROC value shift relative to the enabled pipeline numbers was also examined to show how pipeline quantity affects NN performance. Experimental results demonstrated that NN performance was maximized on average with three to five enabled pipelines (Fig. 1). At six or more, performance gradually dwindled as enabled pipeline numbers increased. For further discussion, we visualized the critical locations in the input image when the model was based on the best pipeline combination (Fig. 2) The heat maps establish that the NN diagnoses the patient as active by extracting information from the medial rectus muscle in CO2 and the central part of the superior and inferior rectus muscles in SA_R.

## Discussion

This study introduced a NN that can evaluate TAO patient activity using orbital CT image with 0.871 AUROC, 0.782 accuracy, 0.786 sensitivity, and 0.779 specificity score. As a 0.8 AUROC or higher is considered good^16^, the proposed NN effectively assist in ascertaining activity and treatment plans for TAO patient. However, this performance could be improved for practical applications. Our results were slightly lower than the 0.922 AUC from a previous study that evaluated TAO patient activity by applying a NN to an orbital MRI^14^, potentially due to NN performance variability or MRI’s sharper resolution. CT is deemed inferior to MRI regarding image detail, as it is unclear whether the image purely reflects acute inflammation in orbital tissues. Nevertheless, the rarity of studies that have evaluated TAO patient activity with orbital CT images highlights the need for improved performance and this study’s significance.

The proposed NN outperformed ConvNeXt, a comparative model, in all performance comparisons and exhibited significantly higher AUROC and sensitivity scores than CSPDenseNet. As the proposed NN is configured to allow multiple slices to be passed through multiple heads simultaneously, it can extract and fuse vital information to improve performance. In addition, most existing NNs were primarily designed to receive a single image (RGB or grayscale). However, learning three-dimensional (3D) CT sequencing’s detailed anatomical information is challenging for these architectures. Furthermore, the enable or disable pipeline procedure in the ablation study may contribute to the improvement by automatically modifying the model to receive only essential information. Through these two approaches, we constructed a framework capable of accurately assisting TAO activity assessments with improved performance over comparative models.

The algorithm in our ablation study also assists in determining significant slice and tissue changes in CT image interpretation. Our results indicated that CO2 (the coronal slice located 1/2 distance between the largest eyeball and orbital exit slices) and SA_R (the sagittal slice for the right eye) were selected most frequently during the early procedure stages. Excessive CT slices diminish NN performance due to unnecessary information. Therefore, identifying the minimum CT slice amount with the greatest efficacy is necessary for improving NN performance. Previous studies that applied NNs to CT also selected and analyzed a few CT slices rather than analyzing all CT images^17,18^. 3D CT reconstruction may help preserve as much necessary information as possible.

Interestingly, we determined that incorporating age and sex did not notably improve NN performance. Age and sex are standard patient information, and epidemiological studies have established that TAO is more common in women, whereas older men are more likely to suffer from worse clinical features^15,19^. Therefore, the AI models also analyzed this data as it could potentially enhance their understanding and analysis of TAO patient activity. Nevertheless, age and gender were not conducive to determining TAO activity in this study. Although the exact reason remains unclear, CT images may already incorporate data more efficacious for determining TAO activity than age or sex. As such, TAO patient activity can likely be judged in practical settings without this information if clinical characteristics such as proptosis, diplopia, eyelid, and anterior segment finding are sufficiently provided. Alternatively, age and sex may already be considered in CT images^20,21^. Thus, age and gender data may not influence NN performance. Further research is needed to conclude if supplemental clinical data related to TAO activity can improve NN performance, such as thyroid function tests, thyroid-stimulating hormones receptor antibody levels, smoking status, or clinical photos.

We compared the proposed NN with two conventional NNs to evaluate its performance. CSPDenseNet has recently performed well as a convolutional NN-based model in image classification and is the proposed model’s baseline. In addition, DenseNet-based models are widely used in the medical field regardless of disease or imaging modality^22–24^. ConvNeXt, based on ResNet^25^, is the most modern NN architecture developed capable of reaching Transformer-based classification models’ overwhelming performance. ResNet performance was improved by applying novel CNN technologies, such as changing the stage-compute ratio. In addition, ResNet is the most renowned model in computer vision and medical image analysis^26–28^.

Although designing a NN by selecting all CT slices is theoretically better, surplus information will likely amplify noise and diminish performance. Therefore, only eleven CT slices were selected and analyzed per subject. Researcher favor AX1, the slice with the largest lens in the axial plane, for exophthalmos evaluation using CT scans^29,30^. In addition, CO2 and CO3 coronal slices are 1/2 to 2/3 of the distance from the largest eyeball slice (CO1) to the orbit exit and are frequently recommended for analyzing extraocular muscle thickness or determining compressive optic neuropathy^31,32^. AX2 to AX5, CO2, and CO3 slices are automatically selected from AX1 and CO1, respectively. Furthermore, we selected AX6 for lacrimal gland representation and a sagittal slice expected to show eyelid changes and superior and inferior rectus muscles shifts^33^. These eleven slices were selected as they best reflected CT changes.

Our study has several limitations. First, section bias may exist as the datasets were collected from a single center. In addition, no standardized orbital CT dataset has yet to be established for diagnosing TAO. Furthermore, enlarged datasets are required for additional training and validation. Since convolutional NN-based deep learning requires significant data, larger dataset could improve diagnostic performance. In addition, the CAS assessment can only estimate inflammation and is sometimes insufficient to determine an accurate active status. Therefore, CAS-based NN diagnostic accuracy may be restricted by the limitation of CAS itself. At this stage, NN applications should be limited to complementing CAS rather than replacing it.

In conclusion, we substantiated NNs’ applicability in assessing TAO activity using orbital CTs. The proposed NN can reliably distinguish between active and inactive TAO patients. To our knowledge, there has yet to be a study on NN-based activity evaluation in TAO patients using orbital CT. The utilized code is publicly available at https://github.com/tkdgur658/MTANet. Although we have paid significant attention to TAO activity evaluation, it is essential to improve activity discrimination efficiency further. Our developed NN will assist in accurately diagnosing and evaluating TAO patients and become the basis for smart diagnosis.

## Methods

The Institutional Review Board of Chung-Ang University Hospital approved this study (IRB No, 2029-028-19439), and the informed consent requirement was waived due to its retrospective design. This study was conducted in accordance with the ethical standards outlined in the Declaration of Helsinki.

### Participants

We obtained orbital CT scans (Philips Brilliance 256 Slice CT, Philips Healthcare Systems, Andover, MA, USA) without contrast from TAO patients diagnosed between January 2010 to October 2019. Our study included 432 TAO patients, among whom 144 were diagnosed as active and 288 inactive. TAO patients were diagnosed according to Bartley and Gorman’s criteria^34^. A seven-point modified CAS formula assessed inflammatory activity by assigning a point to each item: retrobulbar pain, eye movement pain, eyelid redness, conjunctival injection, caruncle or plica inflammation, eyelid swelling, and chemosis. TAO patients with a CAS ≥ 3 were classified as active, while patients with CAS < 3 were classified as inactive. The CT scan and inflammatory activity evaluation were performed on the same day. Two ophthalmologists with more than five years of experience in oculoplasty were blinded to patient information for the CT image analysis and clinical inflammatory activity evaluation. The two experts jointly reviewed the images to reach a consensus in case of a disagreement. Patients below age 18, with a previous history of orbital surgery, orbital tumor, blowout fracture, idiopathic orbital inflammation, those with IV steroid treatment or radiation therapy at the time of CT scan taken, and those with incomplete CT scans were excluded.

### Slice selection

Each patient’s orbital CT had 80 to 400 image slices. However, only a few CT slices were selected from axial, coronal, and sagittal planes to improve TAO activity evaluation performance by avoiding diagnostic model confusion due to redundant information. First, we selected the slice with the largest lens in the axial plane (AX1), then slices 3 mm above (AX2) and below (AX3) and 7 mm above (AX4) and below (AX5) AX1. Next, the slice with the largest lacrimal gland was chosen (AX6). In the coronal plane, the slice exhibiting the largest eyeball was selected first (CO1). We then picked slices 1/2 and 2/3 of the distance between CO1 and the orbit exit (CO2, CO3). Next, slices with the largest eyeball in both eyes were selected from the sagittal plane (SA_L, SA_R). Eleven CT slices were selected for each subject: six axial, three coronal, and two sagittal plane slices.

### Data preparation and processing

After slice selection, we performed the Hounsfield Unit windowing process for better structure identification. We used the Pydicom library’s Value of Interest Look Up Table (VOI LUT) function to convert the original CT pixel values into values ranging from 0 to 1. In this study, we set the Window Center to 0 and the Window Width to 200. Next, we segmented identifiable structures from the 11 CT slices, including the eyeball, four rectus muscles, the optic nerve, and the orbital fat with a few exceptions. Because the superior rectus and superior levator palpebrae muscles could not be reliably distinguished from each other, they were segmented together as a single muscle group, namely the levator-superior rectus (SR) complex. The oblique muscles were excluded as they are difficult to distinguish clearly in CT images. Since AX6 was selected to represent the lacrimal glands, only the lacrimal glands were segmented in AX6. In addition, we further segmented the upper eyelid from two sagittal slices due to its TAO relevance. Finally, we segmented the entire orbit in the CO3 slice because it was difficult to distinguish between each four rectus muscles and the optic nerve (Table 4). As a result, we acquired 78 segmentation images from eleven selected slices.

**Table 4:** Segmented structure in CT slices.

CT slices	Criteria
AX1	Orbit, eyeball, MR, LR, ON, orbital fat (both orbit)
AX2	Orbit, eyeball, MR, LR, ON, orbital fat (both orbit)
AX3	Orbit, eyeball, MR, LR, ON, orbital fat (both orbit)
AX4	Orbit, eyeball, Levator-SR complex, orbital fat (both orbit)
AX5	Orbit, eyeball, IR, orbital fat (both orbit)
AX6	Lacrimal gland (both orbit)
CO1	Eyeball, orbital fat (both orbit)
CO2	MR, LR, levator-SR complex, IR, ON, orbital fat (both orbit)
CO3	Orbit (both orbit)
SA_L	Eyeball, levator-SR complex, IR, ON, upper eyelid (left orbit only)
SA_R	Eyeball, levator-SR complex, IR, ON, upper eyelid (right orbit only)

*AX1* slice with largest lens in the axial plane, *AX2* slices 3 mm above AX1 in the axial plane, *AX3* slice 3 mm below AX1 in the axial plane, *AX4* slices 7 mm above AX1 in the axial plane, *AX5* slice 7 mm below AX1 in the axial plane, *AX6* slice with the largest lacrimal gland in the axial plane, *CO1* slice with largest eyeball in the coronal plane, *CO2* 1/2 distance between the CO1 and orbit exit, *CO2* 1/3 distance between the CO1 and orbit exit, *SA_L* largest eyeball in the sagittal plane of left orbit, *SA_R* largest eyeball in the sagittal plane of right orbit, *MR* medial rectus, *LR* lateral rectus, *ON* optic nerve, *SR* superior rectus, *IR* inferior rectus.

### Neural network model

**Figure 3:**
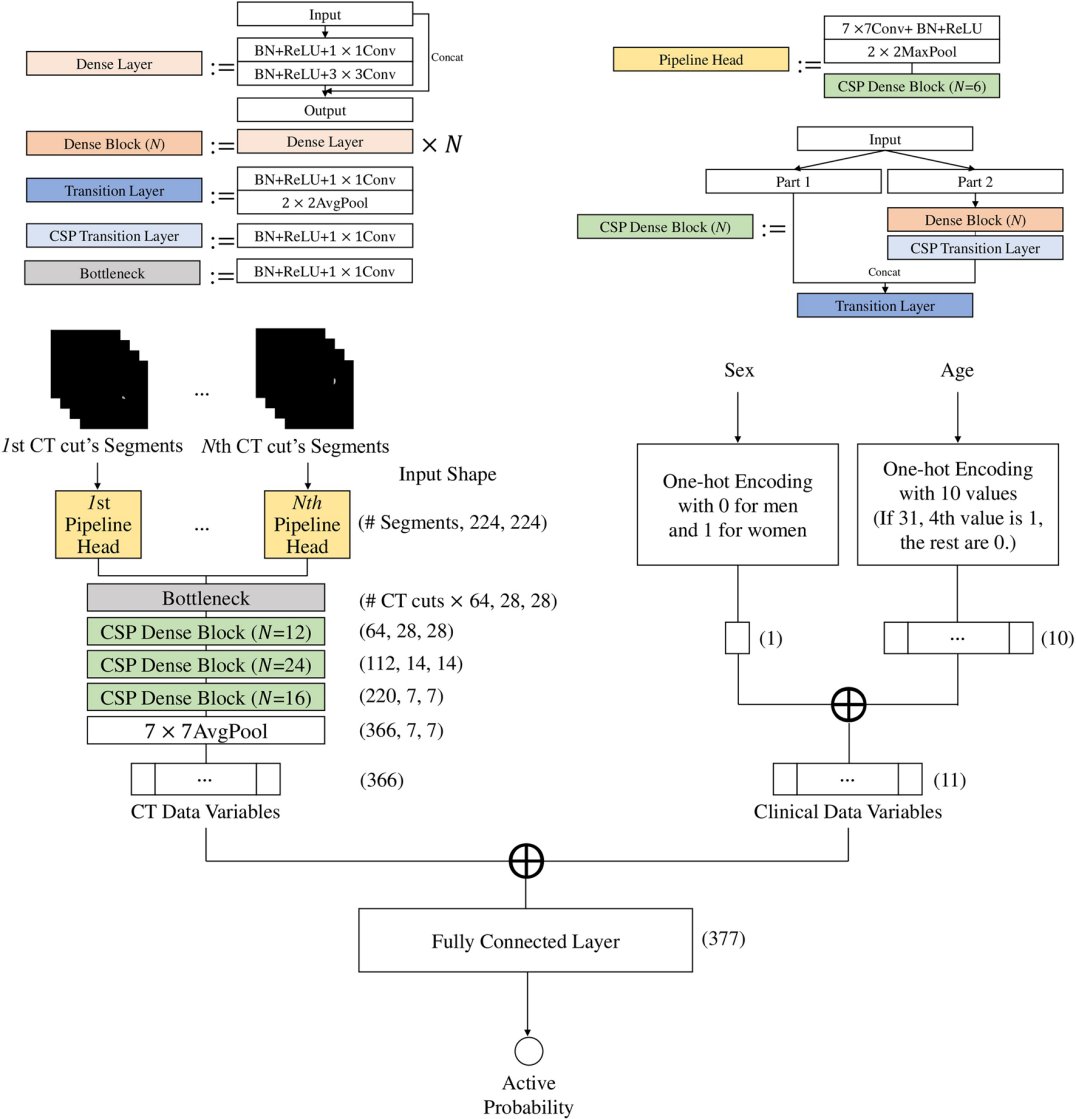
Proposed architecture.

This study defined three considerations to achieve the best TAO activity evaluation performance when devising the proposed NN. First, input slices were chosen from three planes instead of one to capitalize on the advantage of information from multiple views. Second, to exploit possible interactions among identifiable structures in the same slice, the proposed NN processed all segmented images from one slice through a single pipeline; each segmented image was encoded into a channel. Third, we accomplished a pipeline combination ablation study to avoid possible evaluation degradation. Figure 3 illustrates the proposed NN’s overall architecture consisting of pipeline heads, a bottleneck layer, and a model body.

Each CT slice head is an operation sequence constituting 7 × 7 convolution, batch normalization (BN), and ReLU layers and one Cross Stage Partial (CSP) Block. The standard convolution layer output values were calculated with $${y}_{i,j,k}={{w}_{k}}^{T}{x}_{i,j}+{b}_{k}$$ where $${x}_{i,j}$$ was the input value subset centered at $$(i, j)$$, $${y}_{i,j,k}$$ was the output value at $$(i, j)$$ in the $$k$$th feature map, and $${w}_{k}$$ and $${b}_{k}$$ were the $$k$$th filter’s weight vector and bias, respectively. ReLU activation function was defined as $$ReLU(x)=max(x, 0)$$, and the max and average pooling operation output values were $${y}_{i,j,k}=a$$ and $${y}_{i,j,k}=\frac{1}{|{x}_{i,j}|}{\sum }_{a\in {x}_{i,j,k}}a$$ respectively, where $${x}_{i,j,k}$$ was the input value subset centered at ($$i, j)$$ in the $$k$$th input feature map and $${y}_{i,j,k}$$ is the output value at $$(i, j)$$ in the $$k$$th output feature map. The CSP Dense Block was a modified Dense Block that reused vast amounts of gradient information with an improved gradient duplication problem and promising performance^35–39^. The Dense Block was a convolution block with multiple densely connected Dense layers^40^.

The bottleneck layer extracted essential information by compressing head-generated feature maps. Each pipeline head outputs 64 feature maps, generating (number of input CT slices) × 64 feature maps. Next, the proposed NN compresses feature maps based on a 1 $\times$ 1 convolution. The bottleneck layer constitutes a BN layer, a ReLU layer, and a 1 $\times$ 1 convolution. Feature maps passing through the bottleneck were finally compressed into 64 channels. The rest is the model body that processed the compressed feature maps through three CSP Dense Blocks containing 12, 24, and 16 Dense layers. Next, spatial features were compressed into a one-dimensional vector by a 7 × 7 average pooling. The proposed NN concatenated age and sex data with this one-dimensional vector through one-hot encoding, which was considered a pipeline. Finally, the concatenated vector is fed into a fully connected layer and a sigmoid function to output class probabilities. The final active probability $y$ was calculated by

$$y=F(B\left({h}_{1}\left({x}_{1}^{1}, \dots , {x}_{1}^{{n}_{1}}\right), \dots , {h}_{m}\left({x}_{m}^{1}, \dots ,{x}_{m}^{{n}_{m}} \right)\right), c)$$

where ${x}_{i}^{{i}_{k}}$ was the $i$th selected slice’s $j$th segmented image, ${h}_{i}$ was $i$th head, $B$ was a composition function from the bottleneck layer to a 7 × 7 average pooling, $F$ was the fully connected layer, and $c$ was clinical data.

### Ablation study

The proposed NN individually examines input data (11 slices, age and sex) using each pipeline; thus, disabling uninfluential pipelines can distinguish essential information for TAO activity evaluation. To achieve this, we devised a procedure that enables or disables the 13 pipelines by referring to validation performance and estimating the optimal pipeline set. First, the algorithm checks the 13 single-input pipeline performances by only enabling one pipeline at a time to determine the best single-input pipeline. Next, in addition to the best single-input pipeline in the previous stage, a single-input disabled pipeline is enabled to identify the best dual-input pipeline. The algorithm again enables a single-input disabled pipeline with the best dual-input pipeline. The algorithm repeats this procedure until it has considered all 13 pipelines. Finally, the algorithm outputs the final pipeline combination with the best evaluation performance.

### Neural network evaluation

We employed two latest NNs in computer vision to verify the proposed NN’s performance: CSPDenseNet and ConvNeXt. Similar to the proposed NN, the two latest NNs have been slightly modified to receive size 224 $\times$ 224 $\times$ 78 CT slices, age, and sex for a fair comparison. We implemented these models with the Pytorch (1.10.1) library, and all experiments were conducted in a Geforce RTX 3090 24 GB environment. The hyper-parameters were set to a 32 batch size, 30 epochs, the AdamW optimizer, and a 1e-3 learning rate halved every ten epochs by a step-learning rate scheduler. We assessed the three NN performances on six metrics: area under the receiver operating characteristics (AUROC), accuracy, F1-score, sensitivity, specificity, and precision. For training and evaluation, 432 patients were divided into training, validation, and test sets at 0.7, 0.15, and 0.15 ratios extracted through stratified random sampling. The data split process, training, and test were repeated ten times. Furthermore, we conducted additional model comparison experiments based on the selected ten pipeline subsets with the same settings (environment, hyper-parameters, evaluation metrics, data split, repetition). As a result, we could discern essential CT slices out of the collective to aid medical expert interpretation.

### Visual explanation method

Gradient-weighted Class Activation Mapping (Grad-CAM) assisted us in analyzing experimental results^41^ as it generates a visual description regarding the final NN’s decisions. First, Grad-CAM uses gradients for class probabilities to create a coarse score map highlighting essential decision locations. Specifically, a filter’s average gradient values of a specific layer are regarded as that feature map’s importance. Next, each feature map and layer importance are multiplied. Then, all multiplied feature maps are averaged along the channel axis to produce a coarse score map of each region. Since the model receives multiple input images in our visualization, we used the head's feature map and gradient for each slice visualization.

### Statistical analysis

Descriptive statistics are shown as the mean ± standard deviation for continuous variables and the number and percentage for categorical variables. Measured continuous variable comparisons were analyzed using a pair-wise *t*-test. We used Python's open-source library, Scikit-learn, for all statistical analyses of our study; *p-*values $<$ 0.05 were considered statistically significant.

## Data Availability

Datasets used and analyzed during the current study are available from the corresponding author upon reasonable request.
